# Neurosensory Disturbances After Apical Surgery of Mandibular Premolars and Molars: A Retrospective Analysis and Case-Control Study

**DOI:** 10.14744/eej.2021.64326

**Published:** 2021-12-22

**Authors:** Thomas von ARX, Sebastian BOLT, Michael Marc BORNSTEIN

**Affiliations:** 1. Department of Oral Surgery and Stomatology, Faculty of Dental Medicine, University of Bern, Bern, Switzerland; 2. Department of Oral Health & Medicine, University Center for Dental Medicine Basel UZB, University of Basel, Basel, Switzerland

**Keywords:** Altered sensation, apical surgery, mandibular molar, mandibular premolar, neurosensory disturbance, retrospective study

## Abstract

**Objective:**

Apical surgery is an intervention to treat teeth with persistent or recurrent endodontic infection. The proximity of the mental foramen and mandibular canal may pose a risk of altered sensation when performing surgical interventions in the posterior mandible. The objective of this study was to determine the rate of neurosensory disturbances after apical surgery of mandibular premolars and molars. The secondary objective was to evaluate whether the occurrence of altered sensation correlated with the distances from the apex or the periapical lesion to the relevant anatomical structures.

**Methods:**

The charts of patients treated from September 1999 to December 2015 were retrospectively evaluated if an apical surgery had been performed in mandibular premolars or molars, and a minimum period of 1-year follow-up was documented. Patients with trauma or other surgical interventions in the same hemimandible were excluded. Cases with postsurgical altered sensation were defined as the test group. From the pool of unaffected cases, patients were selected to serve as controls. Two- or three-dimensional radiographs of test and control cases were assessed with regard to the shortest distances from the root apices/lesions to the mental foramen or mandibular canal.

**Results:**

The study population included 243 patients with 249 apical surgeries, of which 12.9% led to postoperative neurosensory disturbances. Sensation returned to normal within 22 days on average. The most frequent findings were hyperesthesia or paresthesia. With regard to the treated type of tooth, second premolars resulted more frequently (22.6%) in altered sensation than the other teeth (11.2 to 13.0%; p=0.310). There were no significant differences when comparing test and control cases regarding the measured distances in radiographs from the apex/lesion to the relevant anatomical structures.

**Conclusion:**

Altered sensation may typically occur following apical surgery in posterior mandibular teeth. However, in all cases of the present study, skin sensitivity in the lip/chin region returned to normal. The clinician must be particularly careful when performing apical surgery of second premolars due to the proximity of the mental foramen.

## Introduction

Apical surgery is a well-established treatment option to retain teeth with persisting or recurrent periapical infection. Apical surgery, also known as endodontic microsurgery, is considered a safe intervention with good long-term prognosis (1-3). However, as surgery requires flap elevation, osteotomy, and curettage, a risk to adjacent structures is present. Such structures include neighbouring roots, blood vessels, nerves, the nasal floor, and the maxillary sinus. In addition, in the mandible, apical surgery of premolars or molars may cause damage to the neurovascular content of the mental foramen (MF) or mandibular canal (MC). Consequently, the patient may have temporary or permanent altered sensation in the lower lip and/or chin region. The altered sensation could include dysesthesia, paresthesia, hypesthesia or hyperesthesia. Such sensitivity changes may result in irritation of the lip or drooling, eating disorders, speech problems, depression, and eventually may have medicolegal consequences (4, 5).

**Highlights**

• Apical surgery of mandibular premolars and molars may result in neurosensory disturbances of the skin in the lower lip or chin area.
• The rate of altered sensation in the present study was 12.9%.
• All cases with neurosensory disturbances returned to normal within 22 days on average.
• The highest rate of altered sensation was noted after apical surgery of second premolars.

The assessment of healing in apical surgery has traditionally been on the radiographic assessment of new bone formation within the former bone defect and around the cut root end. Other studies have addressed soft tissue healing or postsurgical quality of life in conjunction with apical surgery (6). In contrast, data about neurosensory changes following apical surgery have only been reported occasionally. A study limited to apical surgery of molars reported an incidence of lower lip sensory deficit in 20-21% of 738 treated mandibular molars (7). Another study including 143 periradicular surgeries in the posterior mandible described an incidence of 10.4% of altered sensation (8). In a recent retrospective analysis of 13 mandibular premolars and 50 mandibular molars treated with apical surgery, altered lip/chin sensation was observed in 14.3% of cases (5).

The primary objective of this retrospective analysis was to determine the rate of neurosensory disturbances after apical surgery of mandibular premolars and molars. The secondary objective was to evaluate a possible correlation between the occurrence of altered sensation and the distances from the apex or periapical lesion to the relevant anatomical structures.

## Materials and Methods

Cases were retrospectively selected from a pool of patients who were treated with apical surgery by a single surgeon from September 1999 to December 2015. The study was approved by the institutional review board (Ethics Commission of Canton Bern, Switzerland, KEK approval number 2016-01711). Cases had been evaluated and treated according to the declaration of Helsinki (www.wma.net) with written consent of all patients.

Cases were included when patients were ≥18 years of age, the apical surgery involved a mandibular premolar or molar, and a 1-year follow-up was documented in the patient chart. Exclusion criteria were patients who have had trauma or other surgical interventions in the same hemimandible, or presented preoperative signs of sensitivity changes in the lower lip/chin regions. 

### Surgical technique

Case selection, technique of apical surgery, as well as medications were described in detail in previous publications (9, 10). Therefore, only a brief summary is presented here. All surgeries were carried out in a dedicated surgical room under local anaesthesia and using a surgical microscope. Triangular or trapezoidal flaps with vestibular release incisions were raised. Flaps were retracted with sutures attached to a hemostat to avoid compression of wound retractors. Osteotomy was performed with rotary burs to access the root apices and lesions, respectively. When proximal to the soft tissue bundle arising from the mental foramen, the osteotomy was performed with piezo-driven inserts (Piezosurgery®, Mectron S.p.A., Loreto, Italy) once they became available on the market. Following debridement of the pathological tissue and root-end resection, haemostasis was obtained with aluminium chloride (ExpasylTM, Produits Dentaires Pierre Roland, Merignac, France) and/or ferric sulfate (Stasis®, GingiPak, Camarillo CA, USA). The cut root face was stained with methylene blue and was inspected with a rigid endoscope. Subsequently, root-end cavities were prepared and obturated with one of the following techniques: microtips either driven by sonic, ultrasonic or piezo devices for root-end filling [SuperEBA (Staident International, Staines, UK), MTA (ProRoot^®^; Dentsply Tulsa Dental, Tulsa OK, USA) or BCRRM (TotalFill^®^, Brasseler, Savannah GA, USA)] or with rotary instruments in case of root-end sealing [adhesive composite (Retroplast^®^, Retroplast Trading, Rorvig, Denmark)]. The endoscope was also used to check the root-end cavity as well as the root-end obturation. After thorough wound cleansing, the flap was repositioned and sutured. Sutures were removed after 4 to 7 days, and all patients were recalled after 1 year for a clinical and radiographic re-examination.

### Case evaluation

With respect to the inclusion/exclusion criteria mentioned above, the charts of 243 patients with 264 treated teeth were assessed (Table 1). In patients with multiple teeth treated in the same hemimandible at the same time, only one tooth was further analysed, based on computer-generated randomisation (Excel®, Microsoft Corporation, Redmond WA, USA).

**Table 1.** Study population undergoing apical surgery

Number of patients	Scenario	Number of teeth	Inclusion/exclusion	Number of teeth analysed
223	One single tooth operated	223	All teeth included	223
3	One single tooth operated and later re-operated	6	Both interventions included	6
13	Two teeth operated at the same time in the same hemimandible	26	Only one tooth included (at random)	13
1	Three teeth operated at the same time in the same hemimandible	3	Only one tooth included (at random)	1
3	One single tooth treated in each hemimandible at two different timepoints	6	All teeth included	6
243		264		249

For the subsequent radiographic evaluation, the teeth resulting in altered sensation were defined as the test group. Matching cases from the non-affected patients were identified and classified as the control group (see below).

### Radiographic evaluation

For the evaluation of a possible correlation between the occurrence of neurosensory disturbances and the proximity of relevant anatomical structures – i.e. MF or MC – cases of the test group were identified that had adequate preoperative radiographic depiction of the MF or MC, either by two-dimensional radiography (2D panoramic view) or three-dimensional imaging (cone-beam computed tomography, CBCT). Cases with inadequate radiography, i.e. distorted pictures, images with artefacts, and unclear or incomplete depiction of the MF or MC, were excluded from the radiographic analysis.

### Case-control selection

From the pool of treated cases without postoperative altered sensation, cases matching those with postoperative neurosensory disturbances were selected. The selection was based on the following criteria (order of priority): same type of imaging technique (2D panoramic views or CBCT), same root, same sex, and similar age.

### Technical details with regard to radiographic imaging

Panoramic images were taken with a Cranex D machine (Soredex, Tuusula, Finland). CBCT images were available from 2004, using a 3D-Accuitomo device (Morita, Kyoto, Japan). Field of views ranged from 4×4 to 8×8 cm. Operating parameters were set at 5 mA and 80 kV, and exposure time was 17.5 seconds. The data were reconstructed with slices at an interval of 0.5 mm.

### Radiographic measurements

**Figure 1.**
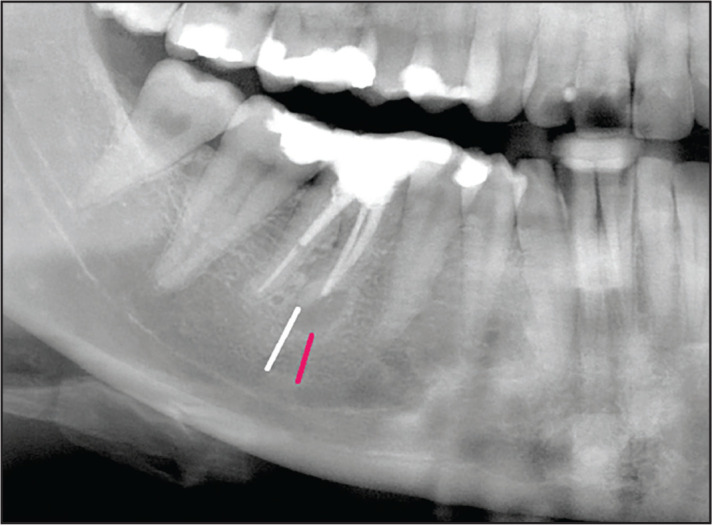
2D panoramic view (cropped image) of the lower right first molar in a 27-year-old female showing the shortest distances from the mesial root apex (white line) as well as from the inferior margin of the lesion to the roof of the mandibular canal (red line)

**Figure 2.**
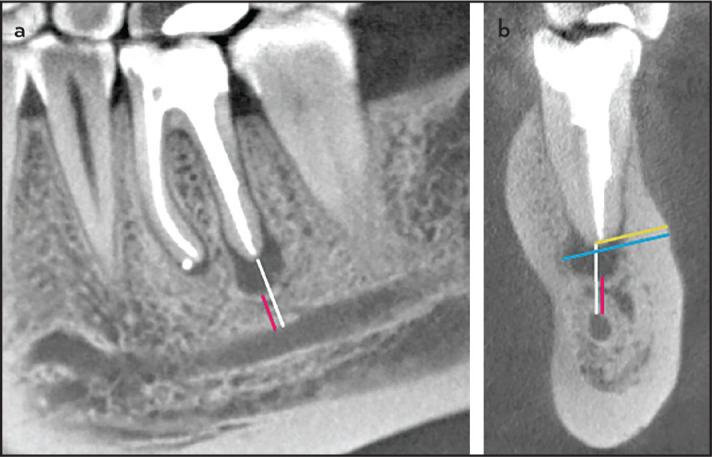
CBCT scans of the lower left first molar in a 34-year-old female. (a) Sagittal cut showing the shortest distances from the distal root apex (white line) as well as from the inferior margin of the lesion to the roof of the mandibular canal (red line). (b) Coronal cut exhibiting the same distances, but also the shortest distances from the buccal bone surface to the apex (yellow line) as well as to the lingual margin of the lesion (blue line) CBCT: Cone-beam computed tomography

Roots of test and control teeth were evaluated with the shortest distances measured from the apex and from the lower margin of the periapical lesion, respectively, to the superior border of the MF/MC, whichever was closer (Fig. 1). In cases where the apex or the lesion projected onto the anatomical structure, the distance received a negative value. In CBCT images, these distances were measured in the sagittal as well as in the coronal plane. On coronal CBCT scans, also the distances from the buccal cortex to the apex and to the lingual margin of the lesion were measured (Fig. 2) (11). 

2D panoramic views were printed out and measurements were done by hand using a ruler. The measured distances were divided by the magnification factor 1.3 of panoramic tomography as indicated by the manufacturer. CBCT images were analysed by using the program i-Dixel (Version 2.5.7, Morita, Kyoto, Japan) with a Dell Precision Tower 5810 workstation (Dell Technologies Inc., Round Rock, TX) and a 27-inch Philips 273E^LH^ LCD monitor with a resolution of 1920×1080 pixels (Philips Electronics N.V, Amsterdam, Netherlands). All measurements were taken twice by a single observer (S.B.) at a minimum interval of 4 weeks. The calculated mean values were utilised for the final analysis.

### Statistical analysis

Mean comparisons were performed by independent t-tests separately. For the associations between two continuous variables, Pearson’s correlations were estimated separately. To investigate the association between the location of the long-axis of the root in relation to the mandibular canal, Fisher’s exact test was performed. For comparing the rates of neurosensory disturbances and the proportion of altered sensitivity among different groups, Fisher’s exact test or Fisher-Freeman-Halton exact test was utilised.

All of the tests performed were two-tailed tests with α=0.05 significance level. The analyses were done using IBM SPSS Statistics for Windows Version 26 (IBM Corp. Armonk, NY, USA).

## Results

The study population (n=243) included 109 males (44.9%) and 134 females (55.1%). The mean age was 49.9 years (range 19 to 78 years). In 32/243 patients (13.2%) or 32/249 interventions (12.9%), temporary neurosensory disturbances were noted after apical surgery of mandibular premolars or molars. The duration of the altered sensation was on average 22 days (median 8 days, range 2 to 207 days). None were reported as permanent. 

Neurosensory disturbances were categorized as hypoesthesia (n=23), paresthesia (n=7), or a combination of the two (n=2). Complete anaesthesia, dysesthesia or hyperesthesia was not recorded. The rate of altered sensation did not differ significantly between males (12.8%) and females (13.4%). In the majority of the affected cases (56.3%), the neurosensory disturbances occurred in the complete supply area of the mental nerve, i.e. the lower lip, corner of the mouth and chin (Table 2).

**Table 2.** Area(s) of neurosensory disturbances

Area(s)	n	%
Lower lip, corner of mouth, chin	18	56.3
Lower lip, corner of mouth	1	3.1
Lower lip, chin	6	18.8
Lower lip	5	15.6
Chin	2	6.3
Total	32	100.0

The distribution of treated teeth, of teeth with subsequent altered sensation, and the calculated percentage rates are summarized in Table 3. In second premolar cases, neurosensory disturbances were noted more often (22.6% out of all second premolars operated) than in the other tooth groups (11.2 to 13.0%). However, this difference was not statistically significant (p=0.310). Furthermore, altered sensation was observed more frequently on the right (16.5%) compared to left sides (8.2%) (p=0.058).

**Table 3.** Distribution of treated teeth and rates of neurosensory disturbances per tooth group

Tooth	Right side	Left side	Total
Distribution of treated teeth (n)			
First premolar	10	13	23
Second premolar	18	13	31
First molar	108	79	187
Second molar	3	5	8
Total	139	110	249
Distribution of teeth with neurosensory disturbances (n)			
First premolar	1	2	3
Second premolar	3	4	7
First molar	18	3	21
Second molar	1	0	1
Total	23	9	32
Rate of neurosensory disturbances per tooth group (%)			
First premolar	10.0	15.4	13.0
Second premolar	16.7	30.8	22.6
First molar	16.7	3.8	11.2
Second molar	33.3	0	12.5
Total	16.5	8.2	12.9

Data about the frequency of altered sensation with regard to gender, age group, and root-end filling are presented in Table 4. None of the analysed parameters exhibited a statistical significance for comparison of subgroups (p<0.05).

**Table 4.** Frequency of neurosensory disturbances per gender, age group, and type of root-end filling

	Number treated	% Treated	Number with altered sensitivity	% Altered sensitivity	P-value
Gender					
Male	112	45	14	12.5	>0.999
Female	137	55	18	13.1
Age group*					
<45 years	81	32.5	14	17.3	0.160
≥45 years	168	67.5	18	10.7
Root-end filling material					
SuperEBA	20	8	2	10	0.074
MTA	161	64.7	15	9.3
Retroplast	63	25.3	14	22.2
BCRRM	4	1.6	1	25
None (resection only)	1	0.4	0	0
All	249	100	32	12.9

With regard to the measured distances (Tables 5, 6), none reached a statistically significant difference when comparing values of the test and control groups. The only statistically significant difference (p=0.005) was noted in the test group for the distance “apex to MF/MC” in 2D panoramic views comparing females (5.1 mm) and males (8.5 mm). A borderline significance (p=0.097) was observed, also in 2D panoramic views of the test group, for the same distance comparing premolars (4.1 mm) and molars (6.8 mm).

**Table 5.** Distances-measured in 2D panoramic views

Study parameter	Subcategory	Test group distance (mm, mean±SD)	Control group mean distance±SD (mm)	P-value
Apex to MF/MC	All	6.2±2.49^a^	7.1±2.56^a^	0.348^a^
	Males (n=5)	8.5±1.80^b^	7.2±3.31^c^	**0.005^b^**
	Females (n=10)	5.1±1.88^b^	7.1±2.31^c^	0.925^c^
	Premolars (n=3)	4.1±1.53^d^	5.5±1.46^e^	0.097^d^
	Molars (n=12)	6.8±2.39^d^	7.5±2.65^e^	0.228^e^
Lesion to MF/MC	All (n=15)	4.8±2.11^f^	5.0±2.87^f^	0.846^f^

Same superscripts denote statistical comparison. Bold P-value denotes a statistically significant difference, MF/MC: Mental foramen/mandibular canal, SD: Standard deviation

**Table 6.** Distances measured in CBCT

Study parameter	Subcategory	Test group mean distance±SD (mm)	Control group mean distance±SD (mm)	P-value
Apex to MF/MC	All	6.6±2.64^a^	6.8±3.07^a^	0.881^a^
	Males (n=7)	8.2±4.01^b^	7.7±2.40^c^	0.074^b^
	Females (n=11)	5.6±1.40^b^	5.9±2.65^c^	0.159^c^
	Premolars (n=3)	7.1±4.05^d^	7.7±3.42^e^	0.831^d^
	Molars (n=15)	6.7±3.01^d^	6.3±2.44^e^	0.362^e^
Lesion to MF/MC	All (n=18)	4.5±3.19^f^	4.5±2.80^f^	0.974^f^
Cortex to apex	All (n=18)	4.6±1.14^g^	5.1±1.55^g^	0.253^g^	Cortex to lesion	All (n=18)	6.8±1.28^h^	7.6±1.73^h^	0.119^h^

Same superscripts denote statistical comparison. CBCT: Cone-beam computed tomography, MF/MC: Mental foramen/mandibular canal, SD: Standard deviation

## Discussion

This study assessed the incidence of altered sensation after apical surgery of mandibular premolars and molars. The strengths of the study include the high number of analysed cases, a single surgeon performed all surgeries, and all interventions were carried out in the same institution. However, the study’s weaknesses are the retrospective nature of the analysis. The data was collected from the charts, and the records could not be verified clinically. For example, if there was no mention of sensitivity change in the patient record, it was assumed that the patient had no altered sensation. Furthermore, sensitivity changes were only determined with the “two-point discrimination” and “sharp vs blunt discrimination” tests without further quantitative, neurological and/or imaging assessments (12). Another shortcoming of this retrospective study was that histology of the removed lesions was not performed routinely. Therefore, a possible correlation of the type of periapical pathology and the occurrence of neurosensory disturbances could not be determined.

**Figure 3.**
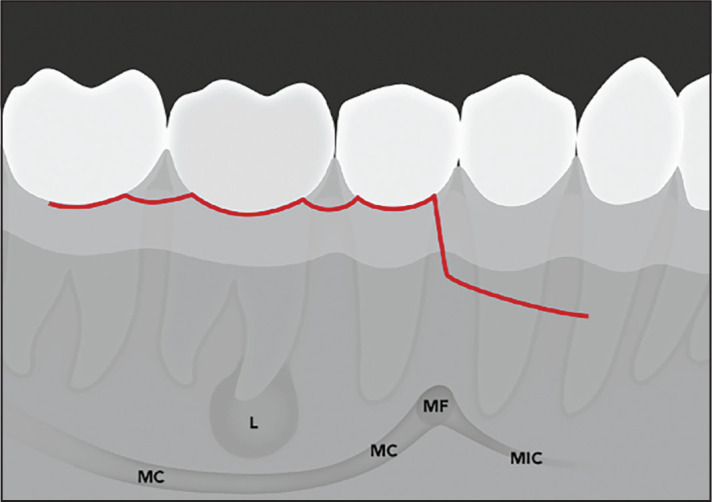
Schematic illustration of a possible flap design to avoid the mental foramen when performing apical surgery of mandibular first molars L: Periapical lesion, MC: Mandibular canal, MF: Mental foramen, MIC: Mandibular incisive canal

The frequency of neurosensory disturbances (12.9%) was similar to previously reported rates of 10.4% (8) and 14.3% (5). In contrast, a marked higher rate (20 to 21%) was described in a large study including only molars (7). Although molars are not necessarily located closer to MF or MC than premolars, the surgical access to molar root apices is more complex. This might result in a larger flap and a longer duration of the surgery. Therefore, either factor could contribute to altered sensation within the supply area of the mental nerve of the lower lip, corner of the mouth, and chin region. In the present sample of patients, special attention was paid to spare the mental nerve with regard to the mesial release incision for apical surgery of molars. Specifically, the initial part of the release incision was placed at the mesial line-angle of the second premolar, then slightly angulated inferiorly but immediately below the mucogingival line. The incision was then directed anteriorly to circumvent the mental foramen with its neurovascular bundle (Fig. 3).

Two vital structures are at risk when performing interventions in the posterior mandible, the MC and the MF. The MC originates from the mandibular foramen at the medial aspect of the ramus. The MC then traverses the body of the mandible. Four typical courses of the MC have linear curve (approximate to a straight line);

• Linear curve (approximate to a straight line);
• Spoon-shape curve (similar to an asymmetric elliptic arc);
• Elliptic arc curve (approximate symmetry);
• Turning curve (unsmooth curve with turning point) (13).

These different MC configurations and varying lengths of roots of the posterior teeth explain that the distances from root apices to MC may show great variability (14). In general, females have shorter distances than males from the relevant root apices to the MC (15-18). With regard to age, root apices of younger individuals tend to be closer to the MC compared to older individuals (16, 19). However, in the present study, age was not found be correlated with the occurrence of altered sensation (Pearson correlation coefficient: 0.564 in test group and 0.527 in control group).

In the axial plane, the MC may also have different configurations, i.e. running along the lingual plate with an anterior sharp turn towards the MF, a stretched S-shaped curve or a linear course through the body of the mandible (20-22). The latter two may pose a higher risk than a lingual position of the MC with regard to the buccal access in apical surgery. However, a separate analysis of the MC position relative to the root apex in the buccolingual plane (coronal CBCT cuts) in the present study did not find any correlation with the occurrence of altered sensation following apical surgery. Therefore, the MC positions in the test and the control groups did not differ significantly.

**Figure 4.**
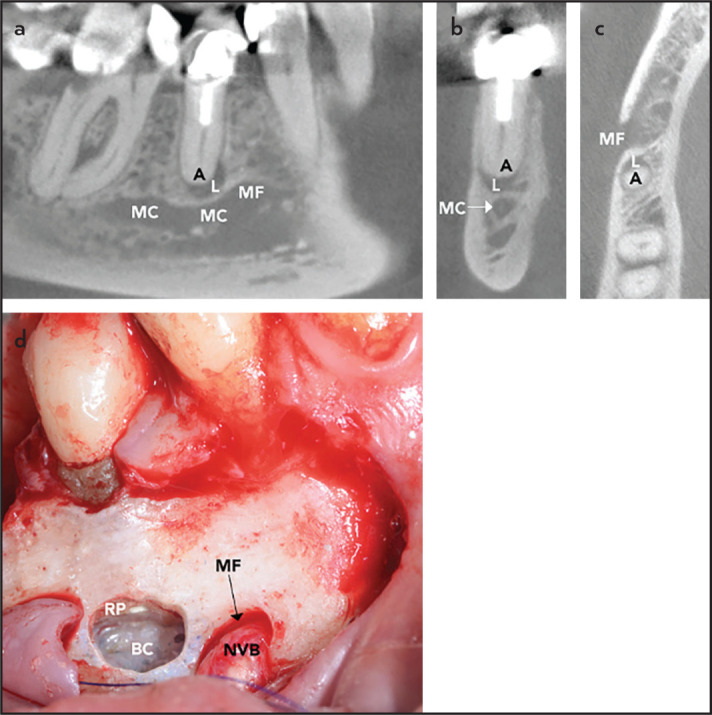
Case illustrating the proximity of the root apex/periapical lesion of the lower right second premolar to the mental foramen in a 66-year-old female. (a) Sagittal cut, (b) coronal cut, (c) axial cut, (d) intraoperative situation following root-end obturation A: Apex of second premolar, L: Periapical lesion, MC: Mandibular canal, MF: Mental foramen, BC: Bony crypt, NVB: Neurovascular bundle, RP: Resection plane

In the region of the premolars, the MC separates into two structures, i.e. the mental canal leading to the MF, and the mandibular incisive canal, extending anteriorly eventually reaching the symphysis. The mental canal is only a short structure, but often shows a “so-called” loop to the MF that is usually directed superoposteriorly. Therefore, the neurovascular bundle of the MF may be closely positioned to the access window of the mandibular premolars (Fig. 4). Minimum distances from the MF to the closest root apex were reported in the literature to range from 0.0 to 2.1 mm (22-24).

Since the MC is located more lingually in the molar region, but then approaches the buccal cortex and ascends to the mental foramen in the second premolar region, apical surgery of second premolars may have an increased risk of postoperative altered sensation. In the present study, second premolars had the highest rate of neurosensory disturbances. Also, Mainkar et al. (5) reported a statistically significant higher rate in premolars (38%) versus molars (8%) (odds ratio 7.19, p=0.014). 

In the present study, altered sensation on the right side (16.5%) was twice as high as on the left side (8.2%). However, no apparent reasons were found to explain this borderline significant difference. However, an operator effect cannot be ruled out as the surgeon was right-handed.

Altered sensation in apical surgery may be caused intraoperatively by direct damage of neural structures with instruments, rotary burs, excessive tension or compression on the flap. While particular caution was exercised to avoid contact of the applied hemostatic agents with the mandibular canal/mental foramen, possible neurotoxic effects could not be ruled out completely. Postoperative haematoma or oedema may also result in altered sensation when they occur in the proximity of the inferior alveolar nerve or mental nerve, respectively. The clinician is therefore advised to exert caution when performing apical surgery in the vicinity of these anatomical structures. In order to avoid compression of the mucoperiosteum by wound retractors, retracting sutures attached to a haemostat are an option. Others have advocated to drill a longitudinal groove into the buccal cortex with the wound retractor engaging this furrow, so the retractor is firmly anchored and does not slip or squeeze the mucoperiosteal flap (25). Location and visualisation of the neurovascular bundle arising from the MF during surgery helps in avoiding inadvertent damage to these vital structures. When root apices or associated periapical lesions project onto the MF or the MC, a CBCT is recommended to assess the three-dimensional relationship and to plan the access window.

## Conclusion

In the present study, 12.9% of apical surgeries of mandibular premolars or molars resulted in postoperative altered sensation. In addition, the frequency of neurosensory disturbances was notably higher in second premolars (22.6%) compared to the other tooth groups (11.2 to 13.0%). It is further noteworthy that skin sensitivity returned to normal in all cases with altered sensation. However, due to the retrospective nature of this analysis, the results must be interpreted with caution.

### Disclosures

**Conflict of interest:** The authors deny any conflict of interest.

**Ethics Committee Approval:** The study was approved by the institutional review board (Ethics Commission of Canton Bern, Switzerland, KEK approval number 2016-01711). Cases had been evaluated and treated according to the declaration of Helsinki (www.wma.net) with written consent of all patients.

**Peer-review:** Externally peer-reviewed.

**Financial Disclosure:** This study did not receive any financial support.

**Authorship contributions:** Concept – T.V.A.; Design – T.V.A.; Supervision – T.V.A.; Funding - None; Materials - T.V.A.; Data collection &/or processing – S.B., M.M.B.; Analysis and/or interpretation – S.B., M.M.B.; Literature search – T.V.A., S.B.; Writing – T.V., M.M.B.; Critical Review – S.B., M.M.B.
